# Enter Salmon: Trophic risk mediates riverine barrier‐crossing behaviours of parr

**DOI:** 10.1111/1365-2656.70301

**Published:** 2026-07-07

**Authors:** Ellen J. Dolan, Gareth Arnott, Jaimie T. A. Dick, Warren Campbell, Ross N. Cuthbert

**Affiliations:** ^1^ School of Biological Sciences, Institute for Global Food Security Queen's University Belfast Belfast UK; ^2^ Department of Agriculture Environment and Rural Affairs Belfast UK

**Keywords:** animal behaviour, fragmentation, freshwater, habitat management, predator–prey interactions, *Salmo salar*

## Abstract

1. Freshwater biota are highly threatened by global stressors, among which fragmentation by artificial barriers has gained increasing attention in recent years. Barriers limit the movements of native species, notably the migrations of keystone fish, such as Atlantic salmon (*Salmo salar*), with ecological and socio‐economic consequences. The severity of barrier impacts on salmon smolt and adult migratory stages has widely overshadowed the effects on juvenile river‐resident stages, despite the importance of habitat availability during these years.
2. We present the first study of crossing behaviours displayed by salmon parr traversing obstacles while considering trophic context‐dependencies.
3. Using semi‐natural flume tanks and a multifactorial design, we examined two of the most common weir designs (vertical and v‐notch) and simulated risk–reward conditions (model predator and chemosensory food resource cues), with approaching, assessing, attempting and crossing behaviours analysed.
4. We found that fish movements throughout the tank were significantly impeded by both barrier designs compared to barrier‐free controls, of which the v‐notch presented a moderately greater challenge.
5. These effects were further mediated by perceived predatory threat, with the presence of a potential predator significantly reducing the probability and latency of fish approaching, reducing the probability of assessing the barrier, as well as shortening the average duration of barrier crossings. Resource presence had minimal effects on the behaviours investigated, except for a significant interaction with predator presence influencing assessment duration.
6. Our study demonstrates that barriers, regardless of their design, place significant limitations on habitats available to parr by reducing overall movement in the system. However, the behaviour of salmon parr around artificial barriers is context‐dependent, highlighting the complexity of fish‐barrier interactions and raising important questions about the potential benefits and unknown consequences of barrier removals within existing ecological networks.

## Graphical abstract

This study provides evidence that artificial riverine barriers alter juvenile Atlantic salmon behaviour in a context‐dependent manner, with predator presence further restricting movement and barrier interactions. By addressing the overlooked impacts of barriers on river‐resident parr stages, our findings contribute to a better understanding of freshwater connectivity and conservation.

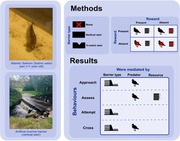

## 1 INTRODUCTION

Freshwater ecosystems are among the most threatened habitats globally and continue to degrade rapidly (Albert et al., 2021). Rivers and lakes provide essential ecosystem services including water supply, food, recreation and hydropower, but exploitation has led to widespread physical modification through dredging, channelisation, bank alteration and fragmentation by artificial barriers (Dudgeon, 2019; Ekka et al., 2020). Artificial barriers are man‐made structures which disrupt the natural abiotic and biotic conditions. Estimates state that half of all rivers are now fragmented, with free‐flowing systems largely confined to remote regions (Grill et al., 2019). Thus, the removal of artificial barriers to restore rivers has gained traction over the last three decades, particularly across Europe and North America (Dolan et al., 2025; Mouchlianitis, 2025).

In Europe, weirs are the most common barrier type and often are the initial focus of barrier removal programmes, due to nonessential uses, being longstanding structures that pose a threat of collapse and their ecological impacts (AMBER Consortium, 2020; Mouchlianitis, 2025). The profile of weirs—including height, slope gradient, construction material and crest design—can vary significantly, each differentially influencing flow regimes, sedimentation dynamics and passage potential by biota, especially fish (Shih et al., 2022). Broad‐crested vertical weirs are one of the most common designs across Europe, where water flows over a flat, wide surface (Badr & Mowla, 2015). The v‐notch weir is a more modern design which is particularly beneficial for improving velocity and turbulence in low flow conditions and has rapidly grown in prevalence since the 20th century (Li et al., 2020; Pospísilík & Zachoval, 2023). While these designs are used for manipulating flow, they can have unintentional and drastically different impacts on fish passage (Baki & Azimi, 2024). Successful navigation of these structures is highly dependent on the swimming capabilities, energy reserves and body size of fish (Egger et al., 2021; Shiau et al., 2020). However, little attention has been paid to the differential effects of barrier types on crossing behaviours, particularly considering keystone species and wider ecological contexts.

Migratory freshwater fish have declined by 76% since 1970, with Atlantic salmon (*Salmo salar*) among the most affected species (Deinet et al., 2024; van Rijssel et al., 2024). In freshwaters, salmon are threatened by climate change, predation, disease and fragmentation (Dudgeon, 2019; Smialek et al., 2021). Targeted efforts to protect populations, such as habitat restoration and stocking initiatives, have been shown to successfully support reproduction and increase population numbers (Lennox et al., 2021). During the upstream migration to their natal spawning grounds, adult salmon typically need to overcome several artificial barriers, often leading to injury, energy loss, delays in migration and death (Algera et al., 2020; Smialek et al., 2021). For those that do make it to headwaters, barriers also alter sediment composition, reducing the suitability of the riverbed for spawning (Newton et al., 2019; Smialek et al., 2021). Barriers can further limit small‐scale movements, drastically reducing accessible habitats of river‐resident juveniles (Buddendorf et al., 2019; Müller et al., 2021). This restriction may intensify intra‐specific competition, reduce foraging success and affect density‐dependent survival (Chen et al., 2023; Fernandez et al., 2024). As a socio‐economically and ecologically invaluable species of the North Atlantic, the restrictions that in‐stream barriers impose on the migration of native Atlantic salmon are a primary motivation behind their removal (Consuegra et al., 2021).

Understanding the attributes and characteristics, not only of the impoundment but also the fish, which mediate successful barrier crossings is vital for targeted barrier removal strategies. Salmon rely on their lateral line, visual and chemosensory systems to detect environmental cues such as obstacles and potential passage routes (Bett et al., 2016; Mogdans, 2019; Mokdad et al., 2023). While most research concerning barrier impacts on salmon has focused on smolt outmigration and adult returning migrations, the parr stage (which spans the first one to 5 years of life) remains overlooked (Ciraane et al., 2025). Juvenile salmon exhibit facultative, bidirectional movements during their territorial parr phase, shifting among microhabitats in response to, for example, flow conditions, foraging needs, predation risk and seasonal habitat changes (Armstrong et al., 2013; Ward et al., 2011; Wells et al., 2025). Detailed behavioural studies at the parr stage are therefore critical to understand how early life history is shaped by fragmentation. Furthermore, ontogenic differences in responses to barriers may be further influenced by wider ecological contexts, with predatory threat and resource reward dynamics potentially mediating behavioural patterns (Deacy et al., 2023; Sortland et al., 2023).

Despite growing interest around salmon conservation and barrier removals, the effect of barriers on river‐resident juvenile stages remains widely overlooked within the context of trophic networks. Thus, the current study aimed to address the knowledge gap around salmon parr responses to in‐stream barriers, through analysis of their crossing behaviours, which were sequentially dissected into approaches, assessments, attempts and crossings, alongside risk‐taking behaviour (Réale et al., 2007; Wilson et al., 1994). We analysed these behaviours in relation to two barrier types (vertical and v‐notch) and examined whether they are further modulated by perceived predation risk or feeding rewards. We posed three predictions: (1) barrier presence will reduce fish movements and alter behaviours associated with successful barrier crossings; (2) perceived predation risk will further reduce fish advancement through habitats; and (3) the presence of a recognisable food source will offset predation risk and increase the frequency and rate of behaviours linked with successful barrier crossings.

## 2 METHODS

### 2.1 Husbandry

Fish collections and experiments were conducted between January and February 2025 at River Bush Salmon Station, Department of Agriculture and Rural Affairs (DAERA, Northern Ireland). A total of 400 one‐year‐old, size‐matched parr (mean length = 11 ± 2 cm) were supplied by the station. Fish were maintained in continually aerated River Bush water under a natural light regime. Holding tanks reflected station standards for husbandry conditions and did not contain additional habitat enrichment. Water was continuously supplied to tanks and dissolved oxygen was monitored by station staff, with an alarm threshold of 5.5 mg L^−1^. During the experimental period, water temperature was recorded routinely (6 ± 0.3°C), and turbidity was visually checked every day prior to trials; trials were not conducted if water clarity reduced markedly, based on visibility of a marked point at the base of the experimental tank (12 cm depth), though no change was observed during the course of the experiments. Each week, 100 parr were transferred to 3 m‐diameter holding tanks (7000 L), from which 36–48 individuals (12 fish per replicate) were randomly selected for trials according to the experimental design (see below). Each fish was used once only. Following experiments, fish were placed in secondary holding tanks before being returned to their original tanks at the station and were not reused. Fish were fed daily with 1.1 g BioMar© pellets per individual, following station protocols. Prior to trials, fish were fed to satiation and then starved for 24 h to standardise motivation.

### 2.2 Ethics Statement

All fish were housed at River Bush Salmon Station for the duration of the experiment, with 180 individuals used in trials. Fish were transferred from holding to experimental tanks using buckets, with minimal air exposure (<10 s per individual). Fish were held for a maximum of 2 weeks before return to the supplier. No overt aggression or visible injury was observed among fish in holding, and any visibly damaged individuals were not used in experiments. This work did not fall under the UK Animals (Scientific Procedures) Act 1986 and was approved by the Queen's University Belfast Faculty Research Ethics Committee (MHLS 24_158).

### 2.3 Barrier design

**FIGURE 1:**
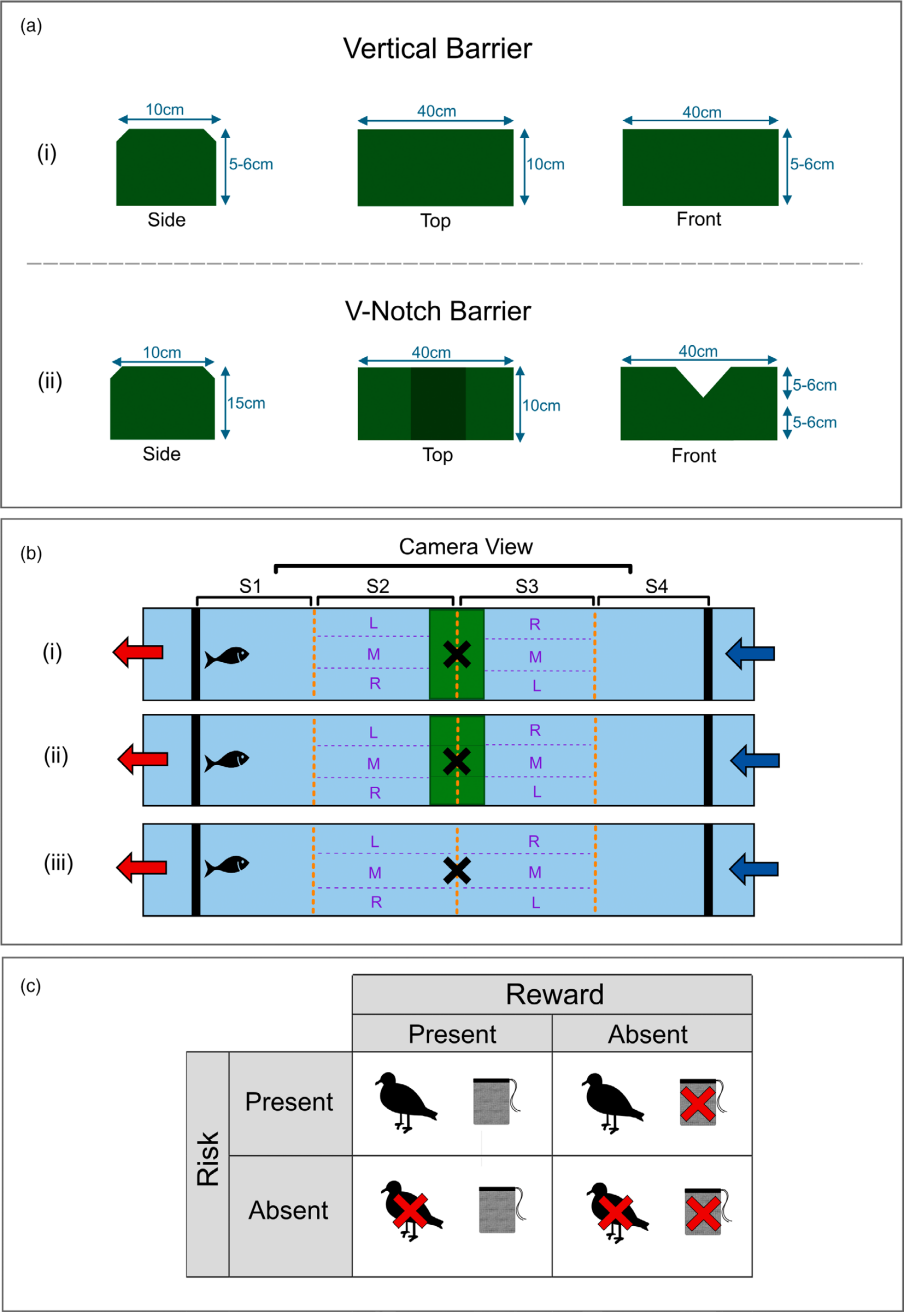
(a) Images of (i) vertical and (ii) v‐notch barriers with the measurement designs of obstacles used in this experiment. (b) Schematic illustrating the experimental set up, including barrier designs (i) vertical, (ii) v‐notch, (iii) none (barrier control). Black line = grate separating acclimation zone (a). Purple dashed lines = left (L), middle (M) and right (R) zones. Orange lines = section (S) markers. Green rectangle = barrier. Blue arrows = water in. Red arrows = water out. Black X = camera placement. Panel (c) depicts the risk–reward matrix, through a black silhouette of a bird representing the predator, and a mesh bag representing the resource.

Barriers simulating two common UK weir types (vertical and v‐notch (Li et al., 2020)) were constructed from sandbags filled with rock and sand and spanned the full width of semi‐natural flume tanks (length = 400 cm; width = 40 cm; depth = 30 cm) and positioned halfway along the tank (1.5 m from upstream and downstream ends). A control without barriers was also included (Figure 1).

### 2.4 Experimental setup

A multifactorial matrix design was created which crossed three barrier types (vertical, v‐notch, none) with predator risk (predator presence versus absence) and resource reward (resource presence versus absence) treatments (Figure 1). Risk treatments were simulated through the presence of a stationary predator, a model gull (22 × 17 cm), which was placed on one randomised left or right side of the barrier per relevant trial. While gulls are not primary predators of wild juvenile parr, avian predators are a threat at later life stages (e.g. migrating smolts; Lothian et al., 2018 and marine adults; Falkegård et al., 2023), and previous studies have demonstrated that juvenile Atlantic salmon exhibit reduced movement and feeding behaviour in response to model predacious birds (Dionne & Dodson, 2002; Metcalfe et al., 1987). Gulls are commonly present around the River Bush salmon station, and thus, the model was unlikely to represent a completely novel visual stimulus, so responses were interpreted as perceived predation risk. Reward treatments consisted of mesh bags containing a known food item (10 g BioMar pellets) placed under inflow at the upstream end of the tank; an empty mesh bag was present during reward absent treatments. Predator and resource cues were added for a 2‐min acclimation period, during which fish were placed at the downstream end of the tank (section one) before the trial began (Figure 1).

Each trial lasted for 10 min and was recorded using GoPro Hero 10 cameras. Three tanks were run simultaneously across four time‐blocks, with one replicate (12 treatments) completed within 2 h. The three barrier conditions were set up concurrently, randomly assigned to one of the three tanks per time‐block within and across replicates; predator and resource treatments were also randomised within and across replicates. Fifteen replicates in total were completed per treatment combination, with a maximum of two replicates per day: one in the morning and a second in the afternoon.

### 2.5 Behavioural data extraction

All data were extracted using Behavioural Observation Research Interactive Software (BORIS). The ethogram included approach, assessment, attempts, crossings and duration spent in each section (Table S1). All behaviours were recorded as state behaviours (i.e. length of time with start and finish time points). Approaches were defined as directed movement towards the barrier from sections two or three, with laterality recorded by dividing the tank into left, middle and right zones (Figure 1). Assessments involved swimming alongside the barrier, with laterality recorded as fish's body orientation relative to the barrier. Attempts were defined as burst swimming oriented towards the barrier, culminating in the head passing over or making contact with the barrier. Attempts were recorded regardless of whether the fish subsequently succeeded in passing the barrier. Crossings were defined as successful passage over the barrier, timed from head entry to tail exit. The probability and latency to leave section one and thus enter section two—an exposed area under perceived potential threat due to closer proximity to the model predator—were used to measure risk‐taking activity.

### 2.6 Statistical analysis

All analyses were conducted in RStudio (v.4.5.0; R Core Team, 2025). Within each fish behaviour, we analysed various aspects including probability, frequency, latency, average duration, and, where applicable, laterality (Table S2). Behavioural responses were analysed using GLMs and GLMMs (*glmmTMB* package), with error distributions matched to each data structure (binomial, Poisson, negative binomial, Gaussian or gamma). Model assumptions were assessed visually and with residual diagnostics (*DHARMa* package).

Barrier type, predator presence, resource presence and their interactions were included as fixed effects. For laterality analyses, fish ID was included as a random effect to account for repeated measures. When the number of observations was small due to low frequency of behaviours (i.e. for crossing latency and duration), the initial model excluded the three‐way interaction. We also note that the initial GLMM assessing frequency of approaches and assessments per side only contained main effects and two‐way interactions between ‘side‐barrier’, ‘side‐predator’ and ‘side‐resource’, as interactions between barrier, predator and resource were already accounted for the corresponding GLM including both sides combined. Analyses of attempts and assessments excluded ‘no barrier’ trials due to the impossibility of behaviour occurrence. Latency and duration models excluded fish that did not perform the behaviour.

Model selection was based on AIC using backward elimination (ΔAIC <2), via the *drop1* function (*base* package), to signal the most parsimonious model (i.e. with minimum AIC). Type III analysis of deviance (*car* package) tested overall effects, with sum‐to‐zero contrasts applied. Significant effects were followed by Tukey‐adjusted pairwise comparisons (*emmeans* package), with back‐transformed estimates where appropriate. Spearman correlations examined associations within each measurement of approaches, assessments, attempts and crossings.

## 3 RESULTS

Barrier conditions significantly influenced attempt frequency and duration, as well as all crossing metrics, with more and longer duration of attempts at v‐notch barriers than vertical, as well as a larger number and faster crossing success without barriers. Predator presence reduced the probability of approach and assessment and shortened crossing duration. The interaction between barrier type and predator further affected approach latency and duration. Resource presence had no main effects but, in combination with predator presence, increased assessment duration. This is a summary of the overarching patterns in the results; further details are provided in subsequent sections (Table 1).

**TABLE 1:** Summary of significant predictors from Type III analysis of deviance on a generalised linear model for the probability, frequency, latency and duration of salmon parr behavioural responses to barriers, predators and resources.

Behaviour	Model	Barrier Type	Predator	Resource
Approach	Binomial (probability)	Ⅹ	✔	Ⅹ
	Poisson (frequency)	Ⅹ	Ⅹ	Ⅹ
	Gamma (latency)	Ⅹ*	Ⅹ*	Ⅹ
	Gamma (duration)	Ⅹ*	Ⅹ*	Ⅹ
Assessing	Binomial (probability)	Ⅹ	✔	Ⅹ
	Negative binomial (frequency)	Ⅹ	Ⅹ	Ⅹ
	Gaussian (latency)	Ⅹ	Ⅹ	Ⅹ
	Gamma (duration)	Ⅹ	Ⅹ*	Ⅹ*
Attempting	Binomial (probability)	Ⅹ	Ⅹ	Ⅹ
	Negative binomial (frequency)	✔	Ⅹ	Ⅹ
	Gamma (latency)	Ⅹ	Ⅹ	Ⅹ
	Gaussian (duration)	✔	Ⅹ	Ⅹ
Crossing	Binomial (probability)	✔	Ⅹ	Ⅹ
	Negative binomial (frequency)	✔	Ⅹ	Ⅹ
	Gaussian (latency)	✔	Ⅹ	Ⅹ
	Gamma (duration)	✔	✔	Ⅹ

*Note*: Due to low frequency of crossings, the three‐way interaction was removed from the initial model for related latency and duration analyses. Ticks and crosses denote significant (*p* < 0.05) and non‐significant (*p* > 0.05) main effects from final models reduced via AIC. Asterisks are used to denote variables further involved in significant interactions.

### 3.1 Barrier approaching

#### 3.1.1 Probability of approach

**FIGURE 2:**
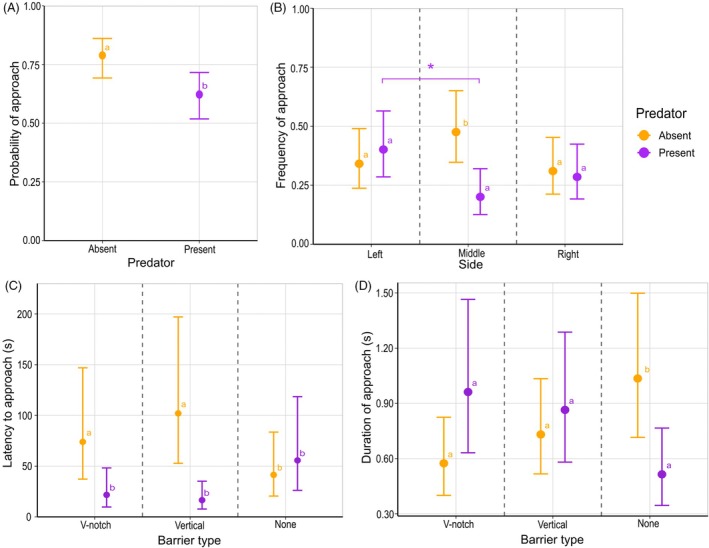
Estimated Marginal Means and 95% Confidence Intervals (CI) of GLM(M) results, with letters representing the statistical results of pairwise comparisons post hoc and asterisks the significant differences among nested levels (i.e. within each *x*‐axis category), where orange represents model predator absent and purple shows present. (A) the probability of a fish to approach the barrier, (B) the frequency of approaches in each zone of the tank with predator conditions, (C) the latency of approaches relative to predator presence nested in barrier conditions and (D) the duration of approaches to the barrier between predator conditions nested within barrier types. Dashed grey lines divide nested levels.

Predator presence significantly reduced the probability of the fish approaching the barrier (Tables 1 and 2, Tables S3 and S4). Salmon exhibited a 79% probability of approaching the barrier in the absence of a predator, compared to 62% in its presence (OR = 2.27 ± 0.77 SE, *z* = 2.43, *p* = 0.015) (Figure 2A).

**TABLE 2:** Results of analysis of deviance on initial and final GLM(M)s of approaching, assessing, attempting, crossing and risk‐taking (i.e. leaving section one) behaviour, showing probability, frequency of occurrences, latency of occurrence, average duration of the behaviours and where relevant, laterality of behaviour.

Behaviour	Measurement	Variable	Final Model		
			LR (χ^2^)	df	*p*‐value
Approach	Probability	**Predator**	**6.080**	**1**	**0.014**
	Frequency	Predator	3.248	1	0.072
	Frequency per side	Fish side	1.765	2	0.414
		Predator	2.688	1	0.101
		**Side:Predator**	**8.403**	**2**	**0.015**
	Latency	Barrier Type	0.289	2	0.865
		**Predator**	**8.718**	**1**	**0.003**
		**Barrier:Predator**	**8.564**	**2**	**0.014**
	Duration	Barrier Type	0.225	2	0.893
		Predator	0.001	1	0.973
		**Barrier:Predator**	**10.099**	**2**	**0.006**
Assessing	Probability	**Predator**	**5.815**	**1**	**0.016**
	Frequency	Barrier Type	1.220	1	0.269
		Predator	1.019	1	0.312
		Barrier:Predator	2.973	1	0.085
	Frequency per side	Side	3.203	1	0.073
	Latency	Barrier Type	0.001	1	0.973
		Resource	3.829	1	0.050
		Barrier:Resource	3.036	1	0.081
	Duration	Predator	0.286	1	0.593
		Resource	0.056	1	0.814
		**Predator:Resource**	**10.520**	1	**0.001**
Attempting	Probability	Barrier Type	3.463	1	0.063
		Predator	3.463	1	0.063
	Frequency	**Barrier Type**	**3.959**	**1**	**0.047**
	Latency	Predator	1.879	1	0.170
	Duration	**Barrier Type**	**4.186**	**1**	**0.041**
Crossing	Probability	**Barrier Type**	**50.959**	**2**	**<0.001**
	Frequency	**Barrier Type**	**43.968**	**2**	**<0.001**
	Latency	**Barrier Type**	**6.755**	**2**	**0.034**
	Duration	**Barrier Type**	**78.807**	**2**	**<0.001**
		**Predator**	**6.847**	**1**	**0.009**
Leaving section one	Probability	**Predator**	**6.415**	**1**	**0.011**
	Latency	NA	‐	‐	‐

*Note*: Final models were selected using backward elimination (ΔAIC <2). Significant results from the final models are in bold.

#### 3.1.2 Frequency of approaches

The final model showed that no variables significantly affected the total number of approaches when approach zones were pooled (Tables 1 and 2, Tables S3 and S4). However, with the approach zone partitioned into left‐middle‐right, barrier type and predator presence had significant effects on the side of the tank that fish approached the barrier on.

In the absence of a predator, fish most frequently approached from the middle zone (mean = 0.48 ± 0.08 SE), followed by the left (0.34 ± 0.06) and right zones (0.31 ± 0.06) [Estimated Marginal Means (EMM) all *p* > 0.16]. In the presence of a predator, fish approached most frequently from the left zone (0.40 ± 0.07), while approaches from the middle zone decreased (0.20 ± 0.05) [(0.20 ± 0.05); EMM(left vs. middle) = 2.000 ± 0.562, *z* = 2.467, *p* = 0.036]. Approaches from the right zone were intermediate (0.29 ± 0.06) and did not differ significantly from either left or middle zones (EMM all *p* > 0.36, Figure 2B).

#### 3.1.3 Latency to approach

Salmon exhibited significantly different latencies to approach within barrier types depending on predator presence (Tables 1 and 2, Tables S3 and S4). Specifically, predator presence significantly shortened the latency to approach both vertical (16.5 ± 6.3 s) and v‐notch (21.7 s ± 8.18 s) barriers, compared to in the absence of a predator (vertical: 102.0 s ± 33.9 s; v‐notch: 73.9 ± 25.6 s). Latency to approach the barrier was similar for no barrier conditions under both the presence (55.7 ± 21.2 s) and absence (41.4 ± 14.7 s) of a predator [vertical: EMM (absent vs. present) = 6.17 ± 3.12, *t* = 3.597, *p* < 0.001; v‐notch EMM(absent vs. present) = 3.41 ± 1.81, *t* = 2.305, *p* = 0.023]. There was no significant effect of predator when there was no barrier (*p* = 0.571, Figure 2C).

#### 3.1.4 Duration of approach

Predator presence tended to mediate the duration of approach to each barrier type (Tables 1 and 2, Tables S3 and S4). When predators were present, average approach duration did not differ significantly among barrier conditions (vertical: 0.865 ± 0.174 s; v‐notch: 0.962 ± 0.204 s; none: 0.515 ± 0.103 s; REM all *p* > 0.16). In the absence of predators, the average approach duration was longest when no barrier was present (1.04 ± 0.19 s), then vertical barriers (0.73 ± 0.13 s), and shortest for v‐notch barriers (0.57 ± 0.11 s). Predator presence significantly reduced approach duration in the no barrier treatment [EMM(absent vs. present) = 2.01 ± 0.55, *p* = 0.012] but had no significant effect for the vertical barrier (*p* = 0.53) and a marginal effect for the v‐notch barrier (*p* = 0.068) (Figure 2D).

### 3.2 Barrier assessment

#### 3.2.1 Occurrence of assessment

**FIGURE 3:**
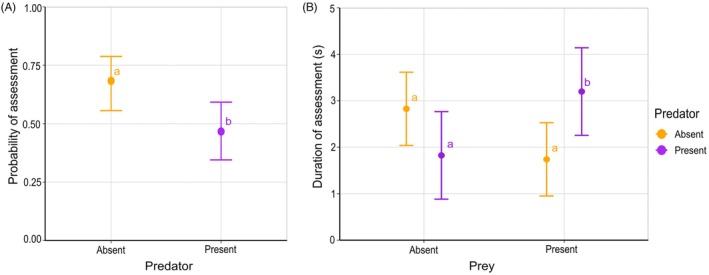
Estimated Marginal Means and 95% CI of GLM results, with significance letters representing the statistical results of pairwise comparison post hoc of the (A) probability and (B) duration of a fish assessing the barrier, where orange represents model predator absent and purple shows present.

The probability to assess the barriers was only significantly affected by the presence of a predator (Tables 1 and 2, Tables S3 and S5). Fish were significantly less likely to assess the barrier in the presence of a predator, with a 47% probability, compared to a 68% probability to assess the barrier in the absence of a predator (OR = 2.47 ± 0.936 SE, *z* = 2.379, *p* = 0.017, Figure 3A).

#### 3.2.2 Frequency of assessment

None of the variables had a significant effect on the frequency of assessments made by the fish (Tables 1 and 2, Tables S3 and S5). However, when examining laterality, fish had a (non‐significant) tendency to assess more times on their left (0.705 ± 0.108) compared to their right (0.525 ± 0.071) side.

#### 3.2.3 Latency to assess

There were no significant effects on latency to assess the barrier (Tables 1 and 2, Tables S3 and S5).

#### 3.2.4 Duration of assessment

Assessment duration was significantly affected by the interaction between predator and resource conditions (Tables 1 and 2, Tables S3 and S5). Mean assessment duration was highest when both predator and resource were present (3.20 ± 0.56) and lowest when predator (1.82 ± 0.32) or resource (1.74 ± 0.25) were present alone, compared to the control (2.83 ± 0.41). Predator presence significantly increased assessment durations when resources were available [EMM(resource vs. both) = 0.54, *p* = 0.045], while all other contrasts were non‐significant (Figure 3B).

### 3.3 Attempting to cross the barrier

#### 3.3.1 Probability of fish attempting

There was no significant relationship between the probability to attempt to cross the barrier with any of the variables (Tables 1 and 2, Tables S3 and S6).

#### 3.3.2 Frequency of attempts to cross

**FIGURE 4:**
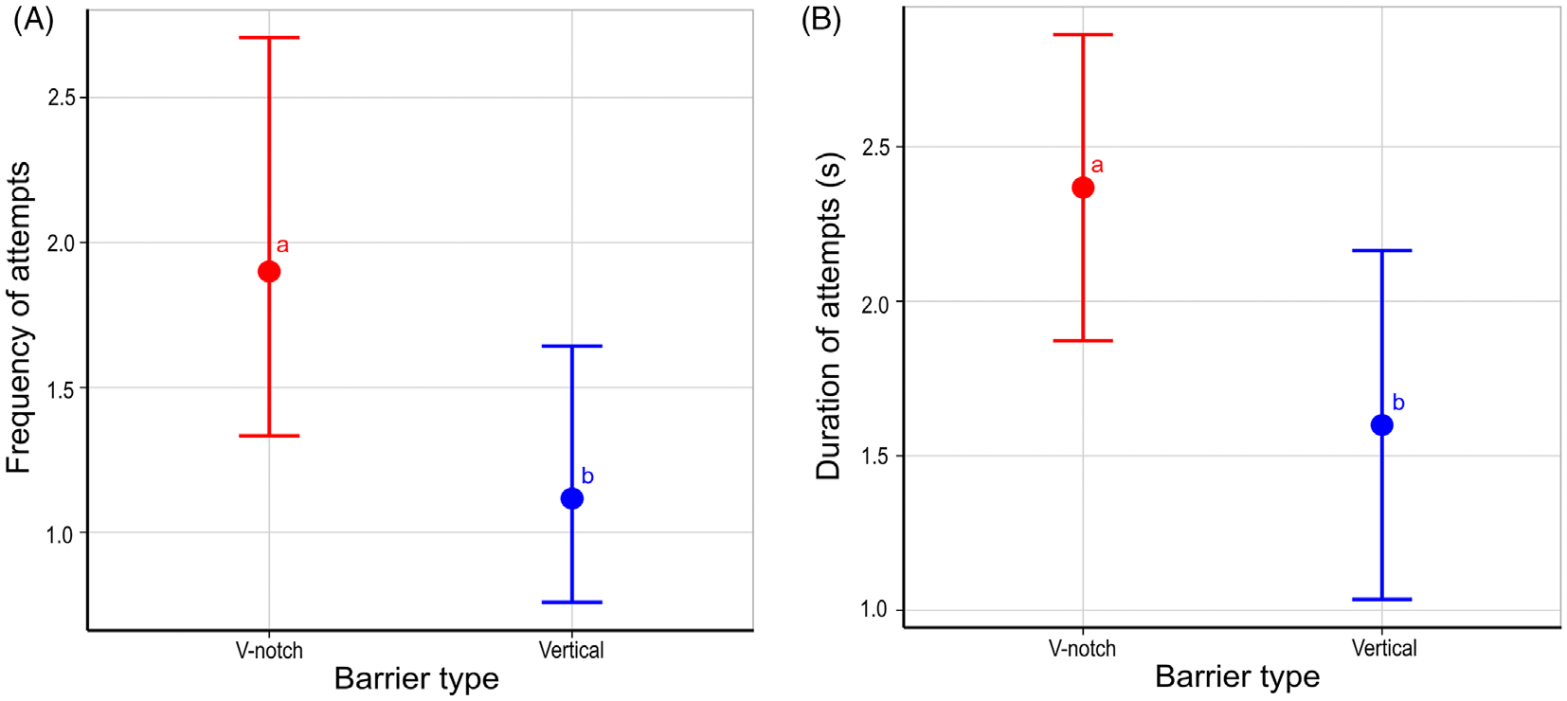
Estimated Marginal Means and 95% CI of GLM results, where the v‐notch barrier is represented by red and vertical by blue, with significance letters representing the pairwise comparison of (A) the frequency of attempts fish undertook per barrier type, and (B) the duration of attempts under each barrier type. All analyses of attempting behaviours excluded trials with no barrier conditions.

Barrier type significantly affected frequency of attempts (Tables 1, 2, S3 and S6). Fish attempted to cross the v‐notch barrier (1.90 ± 0.343) significantly more times than the vertical barrier [1.120 ± 0.220; EMM(vertical vs. v‐notch): 0.588 ± 0.157, *z* = − 1.989, *p* = 0.047] (Figure 4A).

#### 3.3.3 Latency of crossing attempts

We found no significant effect of any of the conditions on the latency to attempt to cross the barrier (Tables 1 and 2, Tables S3 and S6).

#### 3.3.4 Duration of attempts

Barrier type had a significant effect on the duration of attempting to cross the barrier (Tables 1 and 2, Tables S3 and S6). Fish took significantly longer attempting to cross the v‐notch barrier (2.370 ± 0.248 s) than the vertical barrier [1.60 ± 0.282 s; EMM(vertical vs. v‐notch): −0.768 ± 0.376, *t* = −2.046, *p* = 0.045] (Figure 4B).

### 3.4 Barrier crossing

#### 3.4.1 Probability of crossing

**FIGURE 5:**
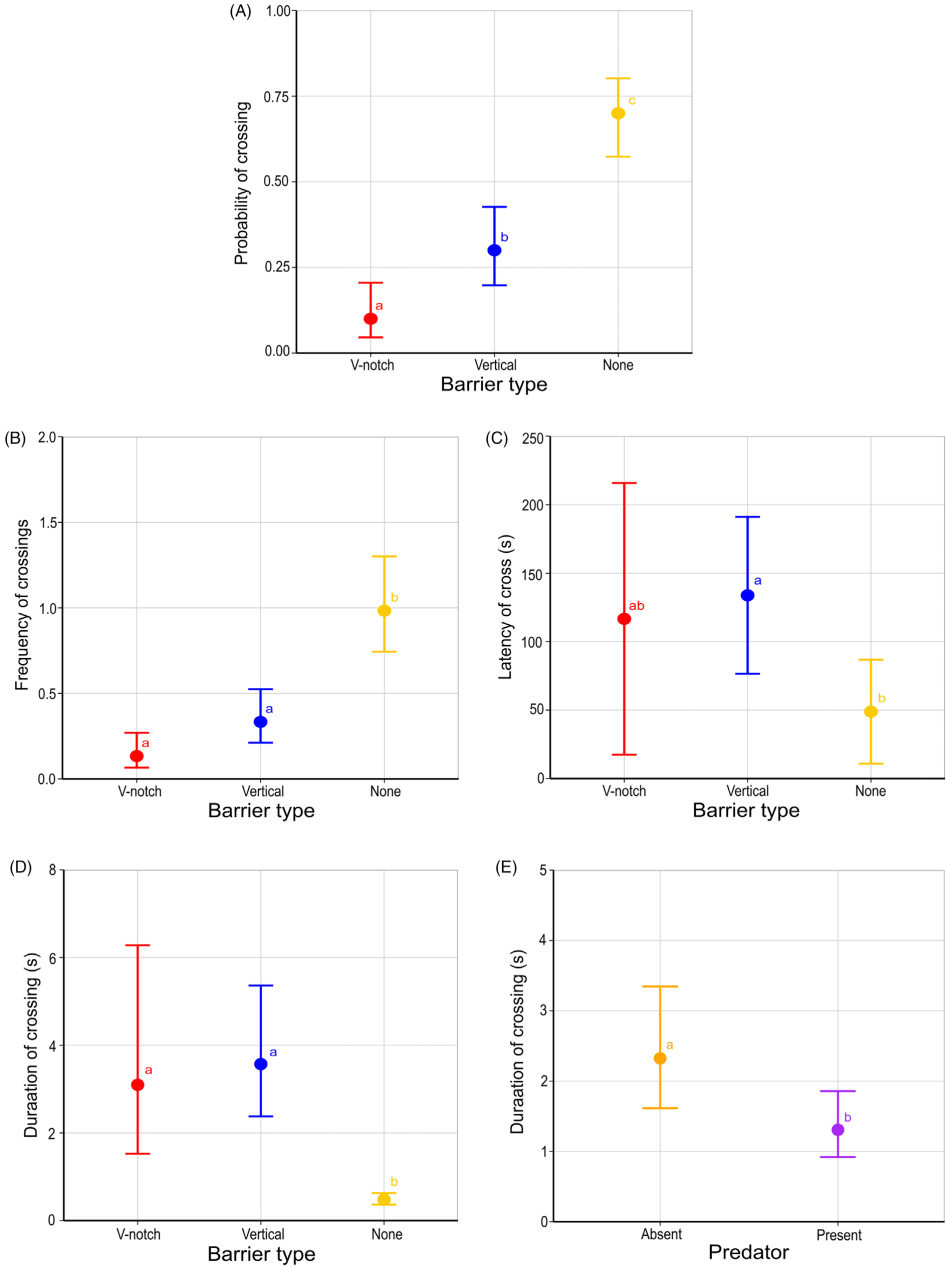
Estimated Marginal Means and 95% CI of GLM results, where the v‐notch barrier is represented by red, vertical by blue and no barrier by yellow; and predator absent conditions are shown in orange and present in purple. Significance letters representing the pairwise comparisons of crossing behaviours including (A) the probability of successful crossings, (B) the frequency of crossings, (C) the latency to cross, (D) duration of crossing among barrier types and (E) duration of crossing within predator conditions.

Barrier type had a significant effect on the probability of a crossing occurring (Tables 1 and 2, Tables S3 and S7). Fish were significantly more likely to cross the tank with no barrier (0.700 ± 0.059) than those under vertical (0.300 ± 0.059) or v‐notch (0.100 ± 0.039) conditions [OR(none vs. vertical) = 5.440 ± 2.170, *z* = 4.253, *p* = 0.001; OR(none vs. v‐notch) = 21.000 ± 10.80, *z* = 5.920, *p* = <0.001]. Fish were also significantly more likely to cross the vertical barrier than the v‐notch barrier [OR(vertical vs. v‐notch) = 3.860 ± 1.980, *z* = 2.625, *p* = 0.024] (Figure 5A).

#### 3.4.2 Frequency of crossing

The frequency of successful crossing the tank was significantly affected by barrier conditions (Tables 1 and 2, Tables S3 and S7). Fish crossed the tank with no barrier (0.983 ± 0.140) significantly more times than the vertical (0.333 ± 0.077) and v‐notch (0.133 ± 0.048) barriers [EMM(none vs. vertical) = 2.950 ± 0.801, *z* = 3.983, *p* < 0.001; EMM(none vs. v‐notch) = 7.380 ± 2.840, *z* = 5.180, *p* < 0.001] (Figure 5B). Fish tended to cross the vertical barrier more times than the v‐notch, though this was not significant [EMM(vertical vs. v‐notch) = 2.5 ± 1.070, *z* = 2.149, *p* = 0.080].

#### 3.4.3 Latency of crossing

Barrier type significantly affected the latency to cross (Tables 1 and 2, Tables S3 and S7). Fish were slower to first crossing of the vertical (133.9 ± 28.7) and v‐notch (116.7 ± 49.6) barriers than no barrier (48.9 ± 19.0) [EMM(none vs. vertical) = −85.0 ± 34.4, *t* = −2.472, *p* = 0.042; EMM(none vs. v‐notch) = −67.8 ± 53.2, *t* = −1.275, *p* = 0.414]. The average latency to cross the v‐notch barrier did not differ from the first successful crossings of the vertical one (Figure 5C).

#### 3.4.4 Duration of barrier crossings

Duration of crossing the barrier conditions was significantly affected by the barrier type and the presence of a predator as separate main terms (Tables 1 and 2, Tables S3 and S7). Fish had significantly longer average crossing durations of the vertical (3.571 ± 0.726 s) and v‐notch barriers (3.096 ± 1.090 s) than without barriers [0.479 ± 0.065 s; EMM(none vs. vertical) = 0.134 ± 0.033, *t* = −8.244, *p* < 0.001; EMM(none vs. v‐notch) = 0.155.2 ± 0.059, *t* = −4.922, *p* < 0.001]. Although the average duration of crossing the vertical barrier was greater than the v‐notch, this was not significant (Figure 5D).

Predator presence also significantly affected the duration of crossings, with fish crossing both barrier conditions significantly faster in the presence of a predator (1.31 ± 0.230) compared to predator absence [2.32 ± 0.423; EMM(absent vs. present) = 1.780 ± 0.383, *t* = −2.669, *p* = 0.009] (Figure 5E).

### 3.5 Risky behaviour

The probability of a fish to leave section one was significantly affected by the presence of a predator only (Tables 2, S3 and S8). The absence of a predator meant that fish were more than two times (OR = 2.38 ± 0.829) more likely to leave section one compared to trials in the presence of a predator. However, for those that did leave, latency to leave section one was not significantly affected by the barrier type, predator or resource presence.

### 3.6 Behavioural associations

**FIGURE 6:**
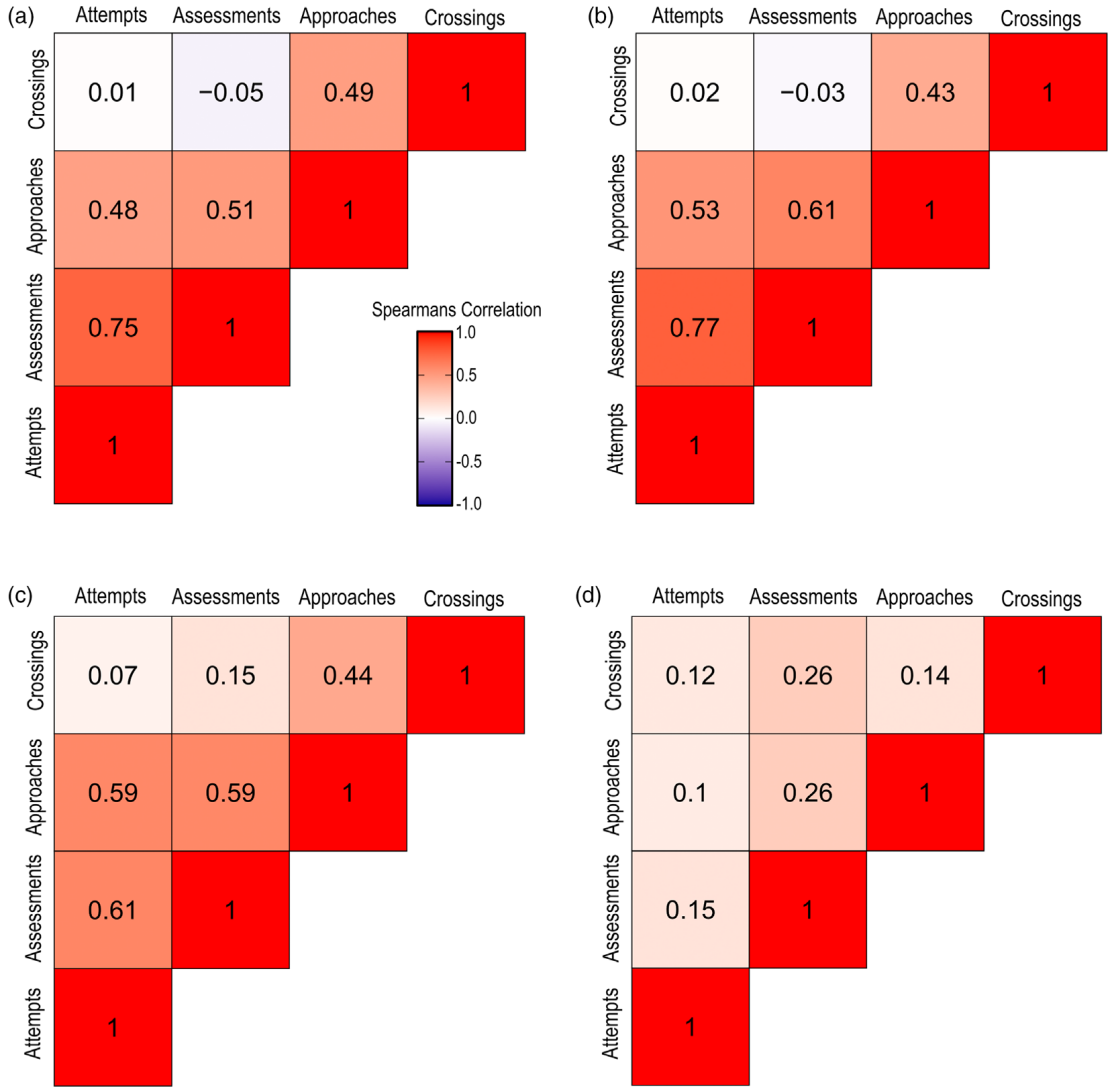
Heatmap of Spearman correlation coefficients between (a) probability, (b) frequency of behaviours, (c) latency and (d) average durations of behaviours. Values represent the strength and direction of associations, with darker colours indicating stronger correlations.

Correlation coefficients showed a significant positive correlation (all *p* < 0.001) between the probability of approaches with attempts, assessments and crossings (Figure 6a). There was a significant positive relationship between assessments and attempts (*p* < 0.001). Whereas no significant correlations were observed between crossings and attempts (*p* = 0.864) or assessments (*p* = 0.467).

Significant positive correlations were observed between most frequencies of behaviours (Figure 6b). The frequency of approaches was significantly positively related to that of attempts, crossing and assessments (all *p* < 0.05). The frequency of attempts was significantly and positively related to assessments (*p* < 0.001). There was no significant correlation found between crossing frequency and attempts or assessments (*p* > 0.05).

There was a significant, positive correlation (*p* < 0.001) between the latency to approach and the latencies to assess, attempt and cross the barrier (Figure 6c). There was again a significant, positive association between the latency to assess and approach the barrier (*p* < 0.001). There was no significant association between the latency to cross and the latency to assess or attempt the barrier (all *p* > 0.300).

Regarding the duration of behaviours, only approaches and assessments (*p* = 0.021) and approaches and crossings (*p* < 0.001) were significantly correlated (Figure 6d). All other behaviours showed no significant correlations.

## 4 DISCUSSION

Artificial barriers alter migration, survival and behaviour of smolts and adult salmon, yet effects on juveniles remain poorly understood (Buddendorf et al., 2019; Shry et al., 2025). Through a multifactorial experimental design, we assessed the behavioural responses of parr to in‐stream barriers under trophic contexts associated with predation risk and resource reward. Barriers reduced the probability and frequency of crossings and increased latency and duration, indicating substantial restriction of longitudinal movement and access to foraging habitats and shelter (Sethi et al., 2022). Often there was minimal variation among barrier types, indicating that altering design or easement of barriers may not be enough to increase habitat availability for this life stage.

Fish typically exploit low‐velocity zones near the banks to conserve energy while moving upstream (Liao et al., 2024). Incorporating this knowledge into barrier design is critical for successful identification and utilisation of passageways (Heneka et al., 2021). Barrier design elicited different crossing success, with fish less probable to cross the v‐notch in comparison to the vertical barrier, indicating lower success when passage near banks is compromised (Larinier, 2002). We reinforce the importance of considering species‐specific sensory and behavioural capabilities in barrier design and remediation strategies, particularly for salmonids that rely on lateral refuge pathways.

Consistent with lateralisation reported in other fish species and hatchery‐reared salmonids, parr tended to assess the barrier using their left side (Axling et al., 2023). Moreover, in the presence of a predator, fish approached the barrier more times on the left than the middle zone of the tank. Predation has previously been shown to influence laterality of fish; however, in the context of barrier crossings, external factors affecting decision‐making remain overlooked (Hulthén et al., 2021; Panizzon et al., 2024). Stronger lateralisation has been linked to migration success (De Russi et al., 2024), suggesting that laterality may influence barrier passage and provide insight into context‐dependent decision‐making. Integrating lateral preferences into fish passage design could improve effectiveness.

Perceived predator risk further mediated barrier effects. Fish were less likely to approach barriers under predation risk, and those that did approached earlier and moved faster. Reduced use of the open, high‐risk mid‐channel zone when the predator was present indicates a behavioural response to predation risk consistent with boldness‐related variation observed among individual salmonids, with risk‐taking typically reduced in exposed areas (Axling et al., 2023; Church & Grant, 2018). Under predation risk, the probability to assess the barrier was reduced and crossing durations were faster, a behaviour that may increase injury risk at high‐velocity structures (García‐Vega et al., 2023). We show that juvenile salmon modify their movements near barriers to evade predators (Dionne & Dodson, 2002). This may exacerbate the limits that physical barriers place on habitat availability by impeding movements despite the availability of a passageway (Dodd et al., 2024; Goerig & Castro‐Santos, 2017).

Although research has traditionally focused on energetic costs, injury and survival during barrier passage (Newton et al., 2019), individual behavioural variation is increasingly recognised as a key determinant of passage success (Mensinger et al., 2021). Consequently, barriers can act as selective filters, favouring individuals with particular behavioural traits, such as boldness and exploratory propensities (Lothian & Lucas, 2021). Over time, this can drive artificial selection and shift gene frequencies towards barrier‐adaptive traits (Goerig et al., 2020; Zarri et al., 2022). Yet, the integration of behaviour into studies of the impacts of artificial barriers remains limited. To our knowledge, while individual fish were exposed only to one treatment, our study has provided the first experimental assessment of salmon parr responses to artificial obstacles in their environment under ecological contexts. Understanding the selective pressures of barriers on certain behaviours is vital, particularly during the juvenile developmental stages.

Barrier‐crossing behaviours were highly correlated, indicating potential behavioural syndromes associated with fragmentation. High association of activity across assessments, attempts, and crossings suggests that certain individuals engage more intensely with barriers. Individual salmon have been shown to exhibit behavioural syndromes—repeatable patterns of behavioural traits—which are context dependent and often altered in anthropogenically modified habitats (Church et al., 2022). Habitat simplification, typically observed near barriers, has been associated with increased boldness and aggression among juvenile fish (Church & Grant, 2018). These behavioural correlations suggest that single metrics may serve as proxies for broader crossing behaviours, though this requires further study. Moreover, research is needed to investigate if experiences with barrier crossings during juvenile stages may condition behavioural syndromes in juvenile salmon and how these might influence their responses to external stressors. However, exploration of how these individual behavioural traits may vary when in conspecific groups is needed. Individual and social behaviour underpins salmonid migration and survival and can alter barrier interactions (Crawford et al., 2025; Mazué et al., 2023). Collective navigation can improve passage discovery but may increase swimming duration and injury risk (Lemasson et al., 2014), while solitary juveniles exhibit erratic movements that may elevate predation risk (Cox et al., 2023). Future work should examine group dynamics and social context in barrier passage.

For effective management and conservation efforts, it is essential that barrier remediation programmes consider juvenile salmon alongside migratory life stages. Importantly, successful passage is highly reliant on barrier design. The reduced performance observed at the v‐notch relative to the vertical barrier suggests that designs concentrating flow through high‐velocity central channels may be less suitable for parr. This aligns with findings from later life stages, where salmonids exhibit higher passage success in fishways that provide low‐velocity marginal pathways or gradual hydraulic guidance towards passage routes (Bunt et al., 2012; Larinier, 2002). Where barriers cannot be removed, retrofits that preserve or create lateral refuge pathways may improve passage.

Hydraulic suitability alone does not determine functional connectivity. Our results demonstrate that perceived predation risk can significantly reduce movement within the environment and alter barrier‐crossing behaviour. This has broader implications for restoration, as artificial barriers may also act as ecological bottlenecks that increase vulnerability to predation. Elevated predation rates have been documented at dams and weirs where fish are delayed or aggregated, creating predictable foraging opportunities for predators (Blackwell & Juanes, 1998; Mensinger et al., 2024). In contrast, natural or nature‐like structures such as beaver dams tend to be more permeable and hydraulically complex, providing multiple passage routes and refuge habitats that may reduce predation risk, particularly for juveniles (Kemp et al., 2012; Larsen et al., 2021; Pollock et al., 2003). Although direct comparisons remain limited, these differences suggest that barrier impacts should be evaluated within a broader ecological framework that incorporates trophic interactions alongside physical connectivity. Accordingly, incorporating habitat cover and reducing predator access (e.g. limiting exposed zones or installing deterrents) around barriers may further enhance passage outcomes when combined with appropriate hydraulic design.

Barrier removal is widely assumed to benefit native populations, yet behavioural responses of juvenile fish and the long‐term consequences remain poorly understood (Bellmore et al., 2019). This study addresses this knowledge gap through assessing the behaviour of parr to obstacles under trophic contexts. However, further work is required to determine how early life interactions with barriers may scale to long‐term population‐level processes. Animal behaviour provides a powerful tool for conservation planning, and failure to account for behavioural responses may undermine restoration efforts (Greggor et al., 2019; Hale et al., 2020). As barrier removal programmes become increasingly important and common for conserving native biota, integrating ecological context and developing a comprehensive understanding of behavioural responses is vital for informing effective and ecologically robust management strategies (Bellmore et al., 2019; Dolan et al., 2025).

## AUTHOR CONTRIBUTIONS

Ellen J. Dolan, Ross N. Cuthbert, Jaimie T.A. Dick and Gareth Arnott conceived the ideas and designed methodology; Ellen J. Dolan collected the data; Ellen J. Dolan analysed the data; Ellen J. Dolan led the writing of the manuscript; Ross N. Cuthbert, Jaimie T.A. Dick, Warren Campbell and Gareth Arnott authors contributed critically to the drafts and gave final approval for publication. Our study was based on a native population of Atlantic salmon, investigated by authors from the region. However, input from all authors spanned various research fields and included expertise in fisheries management. Where possible, our research was discussed with the local public, stakeholders and policymakers to facilitate informative future restoration efforts.

## CONFLICT OF INTEREST STATEMENT

The authors declare no conflicts of interest.

## ETHICS STATEMENT

This work does not fall under the regulations of the UK Animal (Scientific Procedures) Act 1986. Approval was granted by the Queen's University Belfast, Faculty Research Ethics Committee (MHLS 24_158).

## Supporting information


**Table S1.** Descriptions of behaviours analysed.
**Table S2.** Descriptions of measurements of each behaviour.
**Table S3.** Results of analysis of deviance on initial and final GLM(M)s, reduced using backward elimination of terms to achieve the lowest AIC, of approaching, assessing, attempting, crossing and risk‐taking (i.e. leaving section one) behaviour, showing probability, frequency of occurrences, latency of occurrence, average duration of the behaviours, and where relevant, laterality of behaviour. Significant results from the final models are in bold.
**Table S4.** Output from final GLMs and GLMMs regarding the approaching behaviour of fish (*n* = number of fish). For statistical test, *t* values are reported for Gamma models and *z* values are reported for all other models.
**Table S5.** Output from final GLMs and GLMMs regarding the assessing behaviour of fish (*n* = number of fish). For statistical test, *t* values are reported for Gamma models and *z* values are reported for all other models.
**Table S6.** Output from final GLMs regarding the attempting behaviour of fish (*n* = number of fish). For statistical test, *t* values are reported for Gamma models and *z* values are reported for all other models.
**Table S7.** Output from final GLMs regarding the crossing behaviour of fish (*n* = number of fish). For statistical test, *t* values are reported for Gamma models and *z* values are reported for all other models.
**Table S8.** Output from final GLMs regarding risk‐taking activity of fish (*n* = number of fish). For statistical test, *t* values are reported for Gamma models and *z* values are reported for all other models.

## Data Availability

Data and code will be made publicly available on Zonodo.
