# Supplementary material for: Enter Salmon: Trophic risk mediates riverine barrier‐crossing behaviours of parr
Source: J Anim Ecol. 2026 Jul 7;95(8):1419–34. doi: 10.1111/1365-2656.70301 (PMC13439597; doi:10.1111/1365-2656.70301)
Supplement: Supplementary file 1 [file JANE-95-1419-s001.docx]

**Supplementary Information**

**Table S1.** Descriptions of behaviours analysed.

| Behaviour | Description | Modifier(s) |
| --- | --- | --- |
| Approach | Fish were swimming towards the barrier beginning at the middle of section 2 (swimming upstream) or section 3 (swimming downstream) until their head came close to contact with the barrier. | Left, middle or right, respective to the perspective of the fish. |
| Assessment | Fish swimming alongside the barrier, from left to right or vice versa. | Left or right side of fish closest to the barrier. |
| Attempt | Fish actively swimming into or over the barrier structure. | NA |
| Cross | Complete traversing of the barrier, beginning when the head of the fish crested the start of the barrier and ended when the tail was completely over the barrier. | NA |

**Table S2.** Descriptions of measurements of each behaviour.

| Measurement | Description |
| --- | --- |
| Probability | The chance that the behaviour will occur under the described set of conditions, expressed as a number between 0 and 1. |
| Frequency | The number of times a behaviour occurs. |
| Latency | The time until the first occurrence of the behaviour |
| Duration | Length of time over which the behaviour was done, averaged by the number of occurrences. |
| Laterality | The number of times a behaviour occurs per side relative to the eye of the fish. |

**Table S3.** Results of analysis of deviance on initial and final GLM(M)s, reduced using backward elimination of terms to achieve the lowest AIC, of approaching, assessing, attempting, crossing and risk-taking (i.e., leaving section one) behaviour, showing probability, frequency of occurrences, latency of occurrence, average duration of the behaviours, and where relevant, laterality of behaviour. Significant results from the final models are in bold.

| Behaviour | | Variable | Full Model | | | | Reduced Model | | | |
| --- | --- | --- | --- | --- | --- | --- | --- | --- | --- | --- |
|  |  |  | *LR (χ²)* | *df* | *p-value* | *AIC* | *LR (χ²)* | *df* | *p-value* | *AIC* |
| Approaches | Probability | Barrier Type | 0.458 | 2 | 0.795 | 231.555 | - | - | - | 216.11 |
|  |  | Predator | 6.019 | 1 | 0.014 |  | **6.080** | **1** | **0.014 *** |  |
|  |  | Resource | 0.010 | 1 | 0.920 |  | - | - | - |  |
|  |  | Barrier:Predator | 1.106 | 2 | 0.575 |  | - | - | - |  |
|  |  | Barrier:Resource | 0.149 | 2 | 0.928 |  | - | - | - |  |
|  |  | Predator:Resource | 0.010 | 1 | 0.928 |  | - | - | - |  |
|  |  | Barrier:Predator:Resource | 2.481 | 2 | 0.289 |  | - | - | - |  |
|  | Frequency | Barrier Type | 0.534 | 2 | 0.766 | 497.308 | - | - | - | 484.786 |
|  |  | Predator | 3.320 | 1 | 0.069 |  | 3.248 | 1 | 0.072 |  |
|  |  | Resource | 0.289 | 1 | 0.591 |  | - | - | - |  |
|  |  | Barrier:Predator | 0.846 | 2 | 0.655 |  | - | - | - |  |
|  |  | Barrier:Resource | 4.601 | 2 | 0.100 |  | - | - | - |  |
|  |  | Predator:Resource | 0.001 | 1 | 0.972 |  | - | - | - |  |
|  |  | Barrier:Predator:Resource | 0.871 | 2 | 0.647 |  | - | - | - |  |
|  | Frequency per side | Fish side | 1.935 | 2 | 0.380 | 833.659 | 1.765 | 2 | 0.414 | 828.173 |
|  |  | Barrier type | 1.108 | 2 | 0.575 |  | - | - | - |  |
|  |  | Predator | 2.702 | 1 | 0.100 |  | 2.688 | 1 | 0.101 |  |
|  |  | Resource | 0.058 | 1 | 0.810 |  | - | - | - |  |
|  |  | Side:Barrier | 10.031 | 4 | 0.040 |  | - | - | - |  |
|  |  | Side:Predator | 8.395 | 2 | 0.015 |  | **8.403** | **2** | **0.015 *** |  |
|  |  | Side:Resource | 0.745 | 2 | 0.689 |  | - | - | - |  |
|  | Latency | Barrier Type | 0.173 | 2 | 0.917 | 1183.823 | 0.289 | 2 | 0.865 | 1174.186 |
|  |  | Predator | 9.633 | 1 | 0.002 |  | **8.718** | **1** | **0.003 **** |  |
|  |  | Resource | 0.563 | 1 | 0.453 |  | - | - | - |  |
|  |  | Barrier:Predator | 8.093 | 2 | 0.017 |  | **8.564** | **2** | **0.014 **** |  |
|  |  | Barrier:Resource | 0.162 | 2 | 0.922 |  | - | - | - |  |
|  |  | Predator:Resource | 0.843 | 1 | 0.359 |  | - | - | - |  |
|  |  | Barrier:Predator:Resource | 0.273 | 2 | 0.873 |  | - | - | - |  |
|  | Average duration | Barrier Type | 0.331 | 2 | 0.848 | 172.531 | 0.225 | 2 | 0.893 | 163.394 |
|  |  | Predator | 0.001 | 1 | 0.974 |  | 0.001 | 1 | 0.973 |  |
|  |  | Resource | 0.010 | 1 | 0.752 |  | - | - | - |  |
|  |  | Barrier:Predator | 10.163 | 2 | 0.006 |  | **10.099** | **2** | **0.006 **** |  |
|  |  | Barrier:Resource | 1.800 | 2 | 0.407 |  | - | - | - |  |
|  |  | Predator:Resource | 0.001 | 1 | 0.993 |  | - | - | - |  |
|  |  | Barrier:Predator:Resource | 0.036 | 2 | 0.982 |  | - | - | - |  |
| Assessment | Probability | Barrier Type | 0.000 | 1 | 1.000 | 173.595 | - | - | - | 161.831 |
|  |  | Predator | 1.230 | 1 | 0.267 |  | **5.815** | **1** | **0.016 *** |  |
|  |  | Resource | 0.159 | 1 | 0.690 |  | - | - | - |  |
|  |  | Barrier:Predator | 0.000 | 1 | 1.000 |  | - | - | - |  |
|  |  | Barrier:Resource | 0.082 | 1 | 0.775 |  | - | - | - |  |
|  |  | Predator:Resource | 0.086 | 1 | 0.769 |  | - | - | - |  |
|  |  | Barrier:Predator:Resource | 0.044 | 1 | 0.834 |  | - | - | - |  |
|  | Frequency | Barrier Type | 0.747 | 1 | 0.388 | 379.289 | 1.220 | - | 0.269 | 376.355 |
|  |  | Predator | 1.630 | 1 | 0.202 |  | 1.019 | - | 0.312 |  |
|  |  | Resource | 0.747 | 1 | 0.388 |  | - | - | - |  |
|  |  | Barrier:Predator | 2.187 | 1 | 0.139 |  | 2.973 | - | 0.085 |  |
|  |  | Barrier:Resource | 1.139 | 1 | 0.286 |  | - | - | - |  |
|  |  | Predator:Resource | 2.187 | 1 | 0.139 |  | - | - | - |  |
|  |  | Barrier:Predator:Resource | 0.816 | 1 | 0.367 |  | - | - | - |  |
|  | Frequency per side | Side | 3.236 | 1 | 0.072 | 506.085 | 3.203 | 1 | 0.073 | 499.889 |
|  |  | Barrier Type | 0.102 | 1 | 0.749 |  | - | - | - |  |
|  |  | Predator | 2.134 | 1 | 0.144 |  | - | - | - |  |
|  |  | Resource | 0.136 | 1 | 0.712 |  | - | - | - |  |
|  |  | Side:Barrier | 1.775 | 1 | 0.183 |  | - | - | - |  |
|  |  | Side:Predator | 1.443 | 1 | 0.230 |  | - | - | - |  |
|  |  | Side:Resource | 0.229 | 1 | 0.633 |  | - | - | - |  |
|  | Latency | Barrier Type | 0.005 | 1 | 0.939 | 270.148 | 0.001 | 1 | 0.973 | 265.903 |
|  |  | Predator | 0.971 | 1 | 0.325 |  | - | - | - |  |
|  |  | Resource | 3.458 | 1 | 0.063 |  | 3.829 | 1 | 0.050 |  |
|  |  | Barrier:Predator | 0.04 | 1 | 0.842 |  | - | - | - |  |
|  |  | Barrier:Resource | 2.088 | 1 | 0.148 |  | 3.036 | 1 | 0.081 |  |
|  |  | Predator:Resource | 0.297 | 1 | 0.586 |  | - | - | - |  |
|  |  | Barrier:Predator:Resource | 2.067 | 1 | 0.151 |  | - | - | - |  |
|  | Average duration | Barrier Type | 0.636 | 1 | 0.425 | 230.841 | - | - | - | 227.129 |
|  |  | Predator | 0.268 | 1 | 0.605 |  | 0.286 | - | 0.593 |  |
|  |  | Resource | 0.101 | 1 | 0.751 |  | 0.056 | - | 0.814 |  |
|  |  | Barrier:Predator | 1.343 | 1 | 0.247 |  | - | - | - |  |
|  |  | Barrier:Resource | 0.642 | 1 | 0.423 |  | - | - | - |  |
|  |  | Predator:Resource | 10.802 | 1 | 0.001 |  | **10.520** | **-** | **0.001 **** |  |
|  |  | Barrier:Predator:Resource | 0.765 | 1 | 0.382 |  | - | - | - |  |
| Crossing | Probability | Barrier Type | 54.014 | 2 | <0.001 | 203.334 | **50.959** | **2** | **<0.001 ***** | 191.617 |
|  |  | Predator | 0.661 | 1 | 0.416 |  | - | - | - |  |
|  |  | Resource | 1.058 | 1 | 0.304 |  | - | - | - |  |
|  |  | Barrier:Predator | 2.771 | 2 | 0.251 |  | - | - | - |  |
|  |  | Barrier:Resource | 2.507 | 2 | 0.286 |  | - | - | - |  |
|  |  | Predator:Resource | 3.650 | 1 | 0.056 |  | - | - | - |  |
|  |  | Barrier:Predator:Resource | 1.155 | 2 | 0.561 |  | - | - | - |  |
|  | Frequency | Barrier Type | 24.855 | 2 | <0.0001 | 312.379 | **43.968** | **2** | **<0.001 ***** | 302.703 |
|  |  | Predator | 0.951 | 1 | 0.329 |  | - | - | - |  |
|  |  | Resource | 0.636 | 1 | 0.426 |  | - | - | - |  |
|  |  | Barrier:Predator | 3.404 | 2 | 0.182 |  | - | - | - |  |
|  |  | Barrier:Resource | 6.293 | 2 | 0.043 |  | - | - | - |  |
|  |  | Predator:Resource | 0.287 | 1 | 0.592 |  | - | - | - |  |
|  |  | Barrier:Predator:Resource | 3.713 | 2 | 0.156 |  | - | - | - |  |
|  | Latency | Barrier Type | 5.777 | 2 | 0.056 | 818.399 | **6.755** | **2** | **0.034 *** | 813.495 |
|  |  | Predator | 0.216 | 1 | 0.643 |  | - | - | - |  |
|  |  | Resource | 1.064 | 1 | 0.302 |  | - | - | - |  |
|  |  | Barrier:Predator | 2.097 | 2 | 0.350 |  | - | - | - |  |
|  |  | Barrier:Resource | 0.972 | 2 | 0.615 |  | - | - | - |  |
|  |  | Predator:Resource | 2.438 | 1 | 0.118 |  | - | - | - |  |
|  | Average duration | Barrier Type | 184.149 | 2 | <0.001 | 130.044 | **78.807** | **2** | **<0.001 ***** | 124.559 |
|  |  | Predator | 1.986 | 1 | 0.159 |  | **6.847** | **1** | **0.009 **** |  |
|  |  | Resource | 0.22 | 1 | 0.639 |  | - | - | - |  |
|  |  | Barrier:Predator | 2.576 | 2 | 0.276 |  | - | - | - |  |
|  |  | Barrier:Resource | 1.293 | 2 | 0.524 |  | - | - | - |  |
|  |  | Predator:Resource | 0.137 | 1 | 0.711 |  | - | - | - |  |
| Leave section one | Probability | Barrier Type | 3.549 | 2 | 0.170 | 217.033 | - | - | - | 207.070 |
|  |  | Predator | 9.271 | 1 | 0.002 |  | **6.415** | **1** | **0.011 *** |  |
|  |  | Resource | 1.011 | 1 | 0.315 |  | - | - | - |  |
|  |  | Barrier:Predator | 4.474 | 2 | 0.107 |  | - | - | - |  |
|  |  | Barrier:Resource | 2.039 | 2 | 0.361 |  | - | - | - |  |
|  |  | Predator:Resource | 2.093 | 1 | 0.148 |  | - | - | - |  |
|  |  | Barrier:Predator:Resource | 6.027 | 2 | 0.049 |  | - | - | - |  |
|  | Latency | Barrier Type | 0.095 | 2 | 0.953 | 522.360 | - | - | - | 505.066 |
|  |  | Predator | 0.350 | 1 | 0.554 |  | - | - | - |  |
|  |  | Resource | 0.213 | 1 | 0.645 |  | - | - | - |  |
|  |  | Barrier:Predator | 1.401 | 2 | 0.496 |  | - | - | - |  |
|  |  | Barrier:Resource | 0.515 | 2 | 0.773 |  | - | - | - |  |
|  |  | Predator:Resource | 0.374 | 1 | 0.541 |  | - | - | - |  |
|  |  | Barrier:Predator:Resource | 1.216 | 2 | 0.544 |  | - | - | - |  |

**Table S4.** Output from final GLMs and GLMMs regarding the approaching behaviour of fish (*n* = number of fish). For statistical test, t values are reported for Gamma models and z values are reported for all other models.

| Model | *n* | Predictor | Estimate | SE | Test Stat | *p-value* |
| --- | --- | --- | --- | --- | --- | --- |
| Probability  (Binomial GLM) | 180 | **Predator absent (Intercept)** | **1.318** | **0.258** | **5.104** | **<0.001 ***** |
|  |  | **Predator present** | **-0.819** | **0.338** | **-2.427** | **0.015 *** |
| Frequency  (Poisson GLM) | 180 | **Predator absent (Intercept)** | **0.192** | **0.096** | **2.000** | **0.046 *** |
|  |  | Predator present | -0.261 | 0.145 | -1.794 | 0.073 |
| Frequency per zone  (Poisson GLMM) | 180 | **Left zone, Predator absent (Intercept)** | -1.076 | 0.185 | -5.816 | **<0.001 ***** |
|  |  | Middle zone | 0.332 | 0.228 | 1.456 | 0.145 |
|  |  | Right zone | -0.095 | 0.252 | -0.378 | 0.706 |
|  |  | Predator present | 0.163 | 0.244 | 0.668 | 0.504 |
|  |  | **Middle zone:Predator present** | **-1.025** | **0.362** | **-2.833** | **0.005 **** |
|  |  | Right zone:Predator present | -0.246 | 0.356 | -0.692 | 0.489 |
| Latency  (Gamma GLM) | 125 | **No barrier, Predator absent (Intercept)** | **3.724** | **0.355** | **10.506** | **<0.001 ***** |
|  |  | Vertical barrier | 0.901 | 0.486 | 1.854 | 0.066 |
|  |  | V-notch barrier | 0.579 | 0.496 | 1.169 | 0.245 |
|  |  | Predator present | 0.296 | 0.521 | 0.568 | 0.571 |
|  |  | **Vertical barrier:Predator present** | **-2.116** | **0.726** | **-2.914** | **0.004 **** |
|  |  | **V-notch barrier:Predator present** | **-1.521** | **0.744** | **-2.044** | **0.043 *** |
| Average duration  (Gamma GLM) | 125 | No barrier, Predator absent (Intercept) | 0.035 | 0.187 | 0.186 | 0.853 |
|  |  | Vertical barrier | -0.348 | -0.256 | -1.359 | 0.177 |
|  |  | **V-notch barrier** | **-0.589** | **0.261** | **-2.256** | **0.026 *** |
|  |  | **Predator present** | **-0.699** | **0.274** | **-2.549** | **0.012 *** |
|  |  | **Vertical barrier:Predator present** | **0.867** | **0.382** | **2.266** | **0.025 *** |
|  |  | **V-notch barrier:Predator present** | **1.215** | **0.392** | **3.098** | **0.002 **** |

**Table S5.** Output from final GLMs and GLMMs regarding the assessing behaviour of fish (*n* = number of fish). For statistical test, t values are reported for Gamma models and z values are reported for all other models.

| Model | *n* | Predictor | Estimate | SE | Test Stat | *p-value* |
| --- | --- | --- | --- | --- | --- | --- |
| Probability  (Binomial GLM) | 120 | **Predator absent (Intercept)** | **0.769** | **0.278** | **2.771** | **0.006 **** |
|  |  | **Predator present** | **-0.903** | **0.380** | **-2.379** | **0.017 *** |
| Frequency  (Poisson GLM) | 120 | Vertical barrier, Predator absent (Intercept) | 0.383 | 0.225 | 1.702 | 0.089 |
|  |  | V-notch barrier | -0.147 | 0.324 | -0.453 | 0.651 |
|  |  | Predator present | -0.649 | 0.349 | -1.857 | 0.063 |
|  |  | V-notch barrier:Predator present | 0.819 | 0.476 | 1.719 | 0.086 |
| Frequency per fish side  (Poisson GLMM) | 120 | **Left side (Intercept)** | -0.758 | 0.165 | -4.602 | **<0.001 ***** |
|  |  | Right side | -0.296 | 0.165 | 1.790 | 0.074 |
| Latency  (Gaussian GLM) | 68 | **Vertical barrier, Resource absent (Intercept)** | **3.073** | **0.397** | **7.739** | **<0.001 ***** |
|  |  | V-notch barrier | 0.706 | 0.562 | 1.257 | 0.213 |
|  |  | Resource present | -0.085 | 0.554 | -0.154 | 0.878 |
|  |  | V-notch barrier:Resource present | -1.385 | 0.795 | -1.743 | 0.0862 |
| Average duration  (Gamma GLM) | 68 | **Predator absent, Resource absent (Intercept)** | **1.039** | **0.146** | **7.140** | **<0.001 ***** |
|  |  | Predator present | -0.438 | 0.227 | -1.930 | 0.058 |
|  |  | **Resource present** | **-0.486** | **0.206** | **-2.359** | **0.021 *** |
|  |  | **Predator present:Resource present** | **1.047** | **0.321** | **3.264** | **0.002 **** |

**Table S6.** Output from final GLMs regarding the attempting behaviour of fish (*n* = number of fish). For statistical test, t values are reported for Gamma models and z values are reported for all other models.

| Model | *n* | Predictor | Estimate | SE | Test Stat | *p-value* |
| --- | --- | --- | --- | --- | --- | --- |
| Probability  (Binomial GLM) | 120 | Vertical barrier, Predator absent (Intercept) | 0.141 | 0.320 | 0.44 | 0.659 |
|  |  | V-notch barrier | 0.697 | 0.377 | 1.846 | 0.065 |
|  |  | Predator present | -0.697 | 0.377 | -1.846 | 0.065 |
| Frequency  (Negative binomial GLM) | 120 | Vertical barrier (Intercept) | 0.110 | 0.197 | 0.560 | 0.575 |
|  |  | **V-notch barrier** | **0.532** | **0.267** | **-1.989** | **0.047 *** |
| Latency  (Gamma GLM) | 62 | **Predator absent (Intercept)** | **4.553** | **0.310** | **14.679** | **<0.001 ***** |
|  |  | Predator present | -0.674 | 0.479 | -1.408 | 0.164 |
| Average duration  (Gaussian GLM) | 62 | **Vertical barrier (Intercept)** | **1.600** | **0.282** | **5.670** | **<0.001 ***** |
|  |  | **V-notch barrier** | **0.768** | **0.376** | **2.046** | **0.045 *** |

**Table S7.** Output from final GLMs regarding the crossing behaviour of fish (*n* = number of fish). For statistical test, t values are reported for Gamma models and z values are reported for all other models.

| Model | *n* | Predictor | Estimate | SE | Test Stat | *p-value* |
| --- | --- | --- | --- | --- | --- | --- |
| Probability  (Binomial GLM) | 180 | **No barrier (Intercept)** | **0.847** | **0.282** | **3.001** | **0.003 **** |
|  |  | **Vertical barrier** | **-1.695** | **0.398** | **-4.253** | **<0.001 ***** |
|  |  | **V-notch barrier** | **-3.045** | **0.514** | **-5.920** | **<0.001 ***** |
| Frequency  (Negative binomial GLM) | 180 | No barrier (Intercept) | -0.017 | 0.143 | -0.118 | 0.906 |
|  |  | **Vertical barrier** | **-1.082** | **0.272** | **-3.983** | **<0.001 ***** |
|  |  | **V-notch barrier** | **-1.998** | **0.386** | **-5.180** | **<0.001 ***** |
| Latency  (Gaussian GLM) | 65 | **No barrier (Intercept)** | **48.860** | **18.990** | **2.573** | **0.013 *** |
|  |  | **Vertical barrier** | **84.990** | **34.380** | **2.472** | **0.016 *** |
|  |  | V-notch barrier | 67.790 | 53.15 | 1.275 | 0.207 |
| Average duration  (Gamma GLM) | 65 | **No barrier, Predator absent (Intercept)** | **-0.448** | **0.168** | **-2.675** | **0.009 **** |
|  |  | **Vertical barrier** | **2.009** | **0.244** | **8.244** | **<0.001 ***** |
|  |  | **V-notch barrier** | **1.866** | **0.379** | **4.922** | **<0.001 ***** |
|  |  | **Predator present** | **-0.575** | **0.216** | **-2.669** | **0.009 **** |

**Table S8.** Output from final GLMs regarding risk-taking activity of fish (*n* = number of fish). For statistical test, t values are reported for Gamma models and z values are reported for all other models.

| Model | *n* | Predictor | Estimate | SE | Test Stat | *p-value* |
| --- | --- | --- | --- | --- | --- | --- |
| Probability  (Binomial GLM) | 180 | **Predator absent (Intercept)** | **1.444** | **0.270** | **5.353** | **<0.001 ***** |
|  |  | **Predator present** | **-0.866** | **0.349** | **-2.485** | **0.013 *** |
| Latency  (Gaussian GLM) | 129 | **(Intercept)** | **2.436** | **0.149** | **16.330** | **<0.001 ***** |
